# Exosomes of Human Umbilical Cord MSCs Protect Against Hypoxia/Reoxygenation-Induced Pyroptosis of Cardiomyocytes via the miRNA-100-5p/FOXO3/NLRP3 Pathway

**DOI:** 10.3389/fbioe.2020.615850

**Published:** 2021-01-15

**Authors:** Chenyu Liang, Yang Liu, Huifeng Xu, Junling Huang, Yi Shen, Faxiu Chen, Ming Luo

**Affiliations:** ^1^Department of Cardiology, Tongji Hospital, Tongji University School of Medicine, Shanghai, China; ^2^Department of Geriatrics, Tongji Hospital, Tongji University School of Medicine, Shanghai, China; ^3^Department of Geriatrics, Jiangxi Provincial People’s Hospital Affiliated to Nanchang University, Nanchang, China

**Keywords:** miRNA, hypoxia/reoxygenation, human umbilical cord mesenchymal stem cells, exosome, inflammasome

## Abstract

**Background:**

Acute myocardial infarction (AMI) is one of the leading causes of morbidity and death worldwide. Studies have indicated that microRNAs in mesenchymal stem cell (MSC)-derived exosomes are crucial for treating various diseases.

**Methods:**

Human umbilical cord MSC (hucMSC)-derived exosomes (hucMSC-exo) were isolated and used to treat cardiomyocytes that underwent hypoxia/reoxygenation (H/R) injury. Bioluminescence assessment was used to study binding of miRNA to its targeting gene.

**Results:**

We found that H/R decreased the viability of AC16 cells, increased the expression of NLRP3, and activated caspase-1(p20) and GSDMD-N as well as release of IL-1β and IL-18, and such effects were abolished by administration of hucMSC-exo. Administration of exosomes from negative scramble miRNA (NC)-transfected hucMSCs blocked H/R-caused lactate dehydrogenase release, pyroptosis, and over-regulation of NLRP3 and activated caspase-1(p20) and GSDMD-N as well as release of IL-1β and IL-18. More importantly, in comparison to exsomes from NC-transfected hucMSCs, exsomes from miR-100-5p-overexpressing hucMSCs had more obvious effects, and those from miR-100-5p-inhibitor-transfected hucMSCs showed fewer effects. Functional study showed that miR-100-5p bound to the 3’-untranslated region (3’-UTR) of FOXO3 to suppress its transcription. Moreover, overexpression of FOXO3 abolished the protective effects of miR-100-5p.

**Conclusion:**

Enriched miR-100-5p in hucMSC-exo suppressed FOXO3 expression to inhibit NLRP3 inflammasome activation and suppress cytokine release and, therefore, protected cardiomyocytes from H/R-induced pyroptosis and injury.

## Introduction

Acute myocardial infarction (AMI), a major reason for death, is caused by a sudden blockage in blood supply (Mozaffarian et al., 2015; He and Zou, 2020). Myocardial reperfusion is the restoration of coronary blood flow after a period of occlusion (Hashmi and Al-Salam, 2015). However, rapid restoration of blood flow to myocardium may cause additional injury, namely ischemia/reperfusion (I/R) injury (Surendran et al., 2019). Myocardial I/R injury could induce the priming and triggering of the Nod-like receptor protein 3 (NLRP3) inflammasome (Toldo et al., 2018). Upon activation, the NLRP3 inflammasome promotes the maturation of caspase-1, leading to activation of interleukin 1 beta (IL-1β) and IL-18 (Martinon et al., 2002). Inflammasome activation–stimulated secretion of IL-1β and IL-18 induces a pro-inflammatory cell death called pyroptosis, which is associated with a variety of diseases, including autoimmune disease, neurodegenerative disease, cardiovascular disease, cancer, and AIDS (Walle and Lamkanfi, 2016).

Mesenchymal stem cells (MSCs), also called mesenchymal stromal cells, are able to differentiate into various types of cells (Ankrum et al., 2014). MSCs have been attracting researchers’ attention for decades because of their wide-ranging clinical potential (Pittenger et al., 2019). They are among the most commonly used cell types for human disease treatment, including cancers (Lee and Hong, 2017), arthritic diseases (Ruiz et al., 2016), and cardiac disease (Karantalis and Hare, 2015). MSCs can be isolated from different types of tissues, such as the amniotic membrane, chorionic plate, decidua parietalis, adipose tissue, and bone marrow (Mohamed-Ahmed et al., 2018; Wu et al., 2018; Alstrup et al., 2019). Human umbilical cord tissue–derived MSCs (hucMSCs) are MSCs isolated from human umbilical cord. Compared to MSCs from other sources, hucMSCs can be obtained non-invasively because huc tissue is usually abandoned (Zhu et al., 2019).

Study indicates that the benefits of MSCs may be mainly ascribed to paracrine mediators contained in vesicles (Akyurekli et al., 2015). Exosomes are critical in transferring lipids, proteins, and/or RNAs and have been indicated in various physiological and pathological processes (Yeo et al., 2013). Furthermore, MSC-derived exosomes (MSC-exo) have been shown to be effective in treating endometrial stromal cell injury, cell death in myocardial infarction, sepsis, and fetal brain injury after hypoxia-ischemia (Zhu et al., 2018; Wang et al., 2020).

Exosomes are enriched in different types of bioactive molecules, including but not limited to lipids, proteins, and RNAs (Keerthikumar et al., 2016). MicroRNAs (miRNAs), which regulate about 30–70% of human gene expression, make up an important fraction of exosomal content, and they are crucial bioactive molecules in MSC-exo (Ferguson et al., 2018). MSC exosomal microRNAs have been shown to alleviate kidney injury from I/R (Cantaluppi et al., 2012), reduce ischemia-induced cardiomyocyte apoptosis (Feng et al., 2014), and promote neurite remodeling in the ischemic boundary zone of rats with stroke (Xin et al., 2012). However, the role of hucMSC-derived exosomal miRNAs in hypoxia/reoxygenation (H/R)-induced cardiomyocyte injury remains largely unknown.

In the current study, we report that exosomes derived from hucMSCs protect cardiomyocytes against H/R-induced pyroptosis via miRNA-100-5p. A preliminary bioinformatics analysis identified forkhead box O3 (FOXO3), an upstream regulator of NLRP3 (Xu et al., 2020), as a target for miRNA-100-5p. Accordingly, we also examined the possible involvement of FOXO3/NLRP3 in the protective action of miRNA-100-5p.

## Materials and Methods

### Isolation and Culture of hucMSC

This protocol has the approval of the ethical review board of Tongji Hospital of Tongji University. hucMSCs were isolated following the instructions of the Stem Cell Lab, Airlangga University. Cells were grown in minimum essential medium eagle-alpha modification (α-MEM) (Invitrogen, Carlsbad, CA), followed by PBS-washing, fixing, blocking, incubation with FITC or PE-labeled antibodies specific to CD90, CD44, CD105, CD11b, CD34, and CD45 (eBioscience, Supplementary Table S1) and analyzed by BD FACSCalibur (Franklin Lakes, NJ). Mouse IgG was used for a control.

### Isolation and Characterization of hucMSC-Derived Exosomes (hucMSC-exo)

Cell supernatants were centrifuged to remove cell debris and then passed through a 0.22 μm filter. Exosomes were extracted from the cell supernatants with VEX Exosome Isolation Reagent (Vazyme, Nanjing). Final exosomes were resuspended in PBS. Extracted exosomes were checked using transmission electron microscopy (TEM) (MagHelix, Creative Biostructure, Shirley, NY). The marker proteins, CD63, CD9, and Alix were analyzed using Western blot.

### AC16 Cell Culture

Human AC16 cells were purchased from ATCC (Manassa, VA) and cultured in Dulbecco modified Eagle’s medium (DMEM) with 10% fetal calf serum (FCS), and pen-strep at 37°C in a 5% carbon dioxide humidified incubator.

### Exosomes Uptake Experiment

Internalization of hucMSC-exosomes by AC16 cells was detected by staining with PKH26 Dye (Sigma). Exosomes were diluted with Diluent C and incubated with 6 μl PKH26 dye at 25°C following the manufacturer’s protocol. Five minutes later, 3 ml of FBS was added for an extra 1 min of incubation. The mixture was washed with KSFM and centrifuged at 100,000 g for 80 min at 4°C. Supernatants were discarded, and exosomes were resuspended with 1 ml KSFM. Then, 1,000 ng labeled exosomes were administered to AC16 cells (3 × 10^4^ cells/well) in 24-well plates for 24 h at 37°C, followed by fixation and DAPI (6-diamidino-2-phenylindole) staining.

### Hypoxia/Reoxygenation (H/R) Treatment

Cells (1 × 10^6^/well) were loaded into a 96-well plate and cultured in an atmosphere of 1% O_2_ plus 5% CO_2_ for 4 h and then moved to an atmosphere of 20% O_2_ and 5% CO_2_ for 2 h. Cells cultured normally were used for controls.

### MicroRNA Interference and Overexpression

Cells were seeded in a six-well plate (2 × 10^5^ cells/well) for overnight culture, followed by transfection of mimic (50 nM, AACCCGUAGAUCCGAACUUGUG), inhibitor (50 nM, CACA AGUUCGGAUCUACGGGUU), or a negative control scramble miRNA (NC, 50 nM, UUCUCCGAACGUGUCACGUTT) with LyoVec (InvivoGen, San Diego, CA).

### Quantitative Real Time PCR (qRT-PCR)

RNAs were extracted using Trizol (Invitrogen). One microgram of RNA was used to synthesize cDNA with the Revert Aid^TM^ First Strand cDNA Synthesis Kit (Thermo) with special stem-loop primers for miRNA and random primers for gene expression, respectively. qRT-PCR was performed to quantify the expression level of miR-100-5p; NLRP3 and FOXO3 were measured using SYBR Green PCR Master Mix (Invitrogen) on an ABI 7300 thermocycler (ABI, Beverly, MA). U6 or GAPDH was used as a control for miRNA or gene expression, respectively. The primer sequences are listed in Supplementary Table S2. The 2^–ΔΔ*Ct*^ relative quantification method was used to quantify gene expression.

### Protein Isolation and Western Blotting Analysis

Cells were lysed using ice-cold RIPA buffer (Beyotime, Shanghai, China) and proteins were quantified by a BCA kit (Promega); 25 microgram proteins were separated by 10 or 15% gels and electroblotted to nitrocellular membranes (Bio-Rad). After blockage with 3% BSA, membranes were first incubated with first antibodies (Supplementary Table S3) and then incubated with HRP-conjugated secondary antibody (1:2,500, Beyotime). Immunoreactive signals were visualized using ECL chromogenic substrate (Promega). Densitometry analysis was performed with Image J (NIH, Bethesda, MD, United States).

### Cell Viability Assessment

After AC16 cells were cultured and treated, CCK-8 (Jiancheng Bio, Nanjing, China) was loaded and incubated for 3 h in the dark. The optical density (OD) at 450 nm was detected. Cell viability was calculated as cell viability = OD (treatments)/OD (controls) × 100%.

### Hoechst 33342/PI Staining

The treated cells were collected and incubated with Hoechst 33342 and PI (Solarbio, Shanghai, China) for 20 min at 4°C and observed with a fluorescence microscope (Fujifilm).

### Detection of Lactate Dehydrogenase (LDH) Level

The levels of LDH were determined with an LDH assay kit (Beyotime) according to the manufacturer’s protocol and analyzed by a microplate reader.

### Determination of IL-1β and IL-18 Content

The contents of IL-1β and IL-18 in culture medium were measured with Enzyme-linked immunosorbent assay (ELISA) kits (Jiancheng Bio, Nanjing, China).

### Luciferase Assay

The full-length promoter region of NLRP3 (Wei et al., 2016), 3’-untranslated region (3’-UTR) of FOXO3 predicted by miRWalk (Sticht et al., 2018) were inserted into a pGL3 vector (Promega, Madison, WI). The full length of human FOXO3 cDNA was cloned into a pCDNA3.1 vector (Addgene). To study whether miR-100-5p targets FOXO3, 293T cells were transfected with miR-100-5p mimic, inhibitor, or NC and pGL3-FOXO3 3’-UTR plasmid. To further investigate whether miR-100-5p targets 3’-UTR of FOXO3, 293T were transfected by miR-100-5p mimic or NC and pGL3-FOXO3 3’-UTR (WT) or mutant plasmid. For the NLRP3 promoter activity assay, 293T were transfected with pGL3-NLRP3-promoter and pCDNA3.1-FOXO3 (oeFOXO3) or an empty vector. Lipofectamine 2000 reagent (Invitrogen) was used for transfection. Bioluminescence was measured with Luciferase assay kits (Promega).

### Statistical Analysis

Graphpad Prism 6.0 (San Diego, CA) was used in this study. Student’s *t*-test or analysis of variance (ANOVA) was applied to compare data. A *P* < 0.05 was defined as statistically significant.

## Results

### Isolation and Identification of hucMSC-Derived Exosomes

**FIGURE 1:**
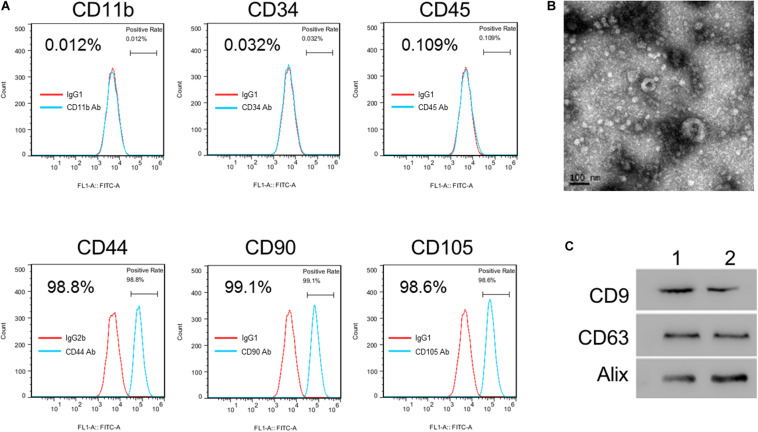
Isolation and identification of hucMSC-exo. **(A)** hucMSC surface markers were detected by flow cytometry analysis. **(B)** Morphology of purified hucMSC-exo. Scale bar: 100 nm. **(C)** Western blotting shows that CD9, CD63, and Alix were expressed in hucMSC-exo isolated from two umbilical cord samples (Panels 1 and 2).

To investigate the potential roles of hucMSC-derived exosomes in H/R-induced injury, hucMSCs were isolated first. Flow cytometric analysis showed the positive expression of CD44, CD90, and CD105 combined with very low expression of CD11b, CD34, and CD45 in isolated hucMSCs (Figure 1A). Next, hucMSC-exo were isolated, and the morphology was checked with a TEM (Figure 1B). Western blot results confirmed the expression of three exosome markers, CD63, CD9, and AliX, in hucMSC-exo from two umbilical cord samples (Figure 1C). Collectively, these results confirm the successful isolation of hucMSCs and hucMSC-derived exosomes.

### hucMSC-exo Protect Against Hypoxia/Reoxygenation-Induced Injury in AC16 Cells

**FIGURE 2:**
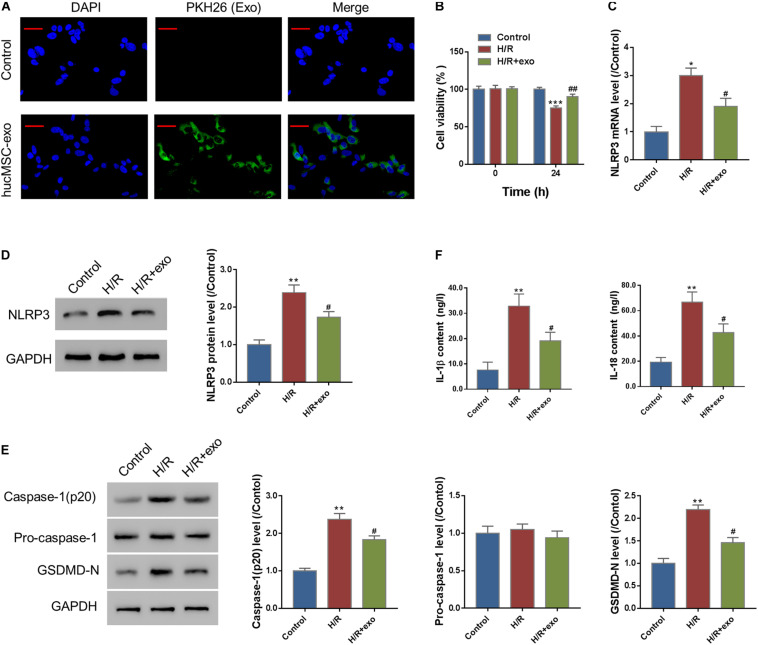
hucMSC-exo protects against H/R-induced injury in AC16 cells. **(A)** hucMSC-exo were internalized by AC16 cells. Scale bar: 50 μm. **(B)** AC16 cells were incubated with hucMSC-exo for 24 h and then subjected to H/R. CCK-8 assay shows that hucMSC-exo significantly increased cell viability, which was suppressed by H/R. **(C,D)** Western blot (**C**, left panel, representative blots; right panel, quantification from three independent experiments) and qRT-PCR **(D)** show that H/R significantly increased NLRP3 expression, which was suppressed by hucMSC-exo. **(E)** Activated caspase-1(p20), pro-Caspase-1, and GSDMD-N were determined by Western blotting. **(F)** The release of IL-1β and IL-18 was determined by ELISA assay. *P < 0.05, **P < 0.01, ***P < 0.001 vs. control; ^#^*P* < 0.05, ^##^*P* < 0.01 vs. H/R.

To analyze the protective effects of hucMSC-derived exosomes on H/R-induced injury, we first performed an exosome uptake assay. The results show that PKH26-stained hucMSC-exo could be internalized by AC16 cells (Figure 2A). Next, AC16 cells were incubated with hucMSC-exo for 24 h and then subjected to H/R challenge. A CCK-8 assay showed that H/R significantly decreased the viability of AC16 cells. In contrast, supplement with hucMSC-exo significantly increased cell viability as compared to the H/R group (Figure 2B). We also checked the effect of hucMSC-exo on NLRP3 expression. As indicated in Figures 2C,D, H/R significantly increased NLRP3 expression as compared to control at both protein and mRNA levels. Administration of hucMSC-exo significantly inhibited NLRP3 expression compared to the H/R-group. Moreover, H/R significantly upregulated the level of activated casp-1(p20) and GSDMD-N (Figure 2E) and the release of IL-1β and IL-18 (Figure 2F), which was suppressed by administration of hucMSC-exo. The data suggest that hucMSC-exo protects AC19 cells from H/R induced NLRP3 inflammasome activation and pyroptosis.

### Exosomal Transferring of miR-100-5p Protects Against Hypoxia/Reoxygenation-Induced Pyroptosis in AC16 Cells

**FIGURE 3:**
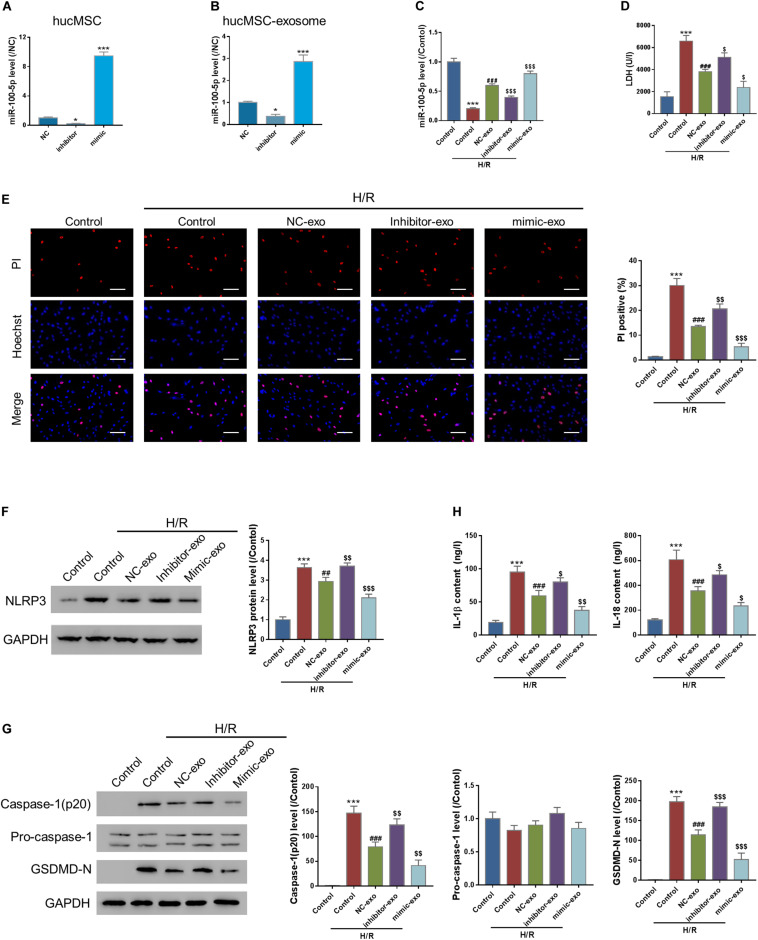
Exosomal transferring of miR-100-5p protects against H/R-induced injury in AC16 cells. **(A,B)** miR-100-5p interference and overexpression in hucMSC and hucMSC-exo. **(C–H)** AC16 cells were incubated with exosomes isolated from hucMSC transfected by NC (NC-exo), miR-100-5p inhibitors (inhibitor-exo), or miR-100-5p mimics (mimic-exo) for 24 h and then subjected to H/R. LDH release **(D)**, pyroptosis rate (**E**, Hoechst 33342/PI staining, Scale bar: 100 μm), protein expression of NLPR3, activated caspase-1(p20), pro-Caspase-1, and GSDMD-N (**F,G**, Western blot analysis, left panel, representative blots; right panel, quantification from three independent experiments), and the release of IL-1β and IL-18 (**H**, ELISA analysis) were determined. *P < 0.05, ***P < 0.001 vs. control; ^##^*P* < 0.01, ^###^*P* < 0.001 vs. Control + H/R; ^$^*P* < 0.05, ^$$^*P* < 0.01, ^$$$^*P* < 0.001 vs. NC-exo + H/R.

MiRNAs are crucial bioactive molecules in MSC-exo. miR-100-5p is the most abundant miRNA in hucMSC-exo (Zhu et al., 2019). To investigate the roles of miR-100-5p in hucMSC-exo, hucMSCs were transfected with either scramble miRNA (NC), miR-100-5p inhibitor (inhibitor), or miR-100-5p mimic (mimic) to silence or overexpress miR-100-5p in hucMSC. Figure 3A indicates that miR-100-5p was successfully silenced or overexpressed in hucMSCs. Then, exosomes were isolated from each group, and miR-100-5p expression was checked. Results show that the exosomal miR-100-5p expression pattern is similar to that in hucMSCs (Figure 3B).

AC16 cells were then incubated with exosomes isolated from the NC (NC-exo), inhibitor (inhibitor-exo), or mimic (mimic-exo) groups for 24 h, followed by H/R challenge. qRT-PCR results show that H/R significantly suppressed miR-100-5p expression compared to the control, which was abolished by supplementation of NC-exo and mimic-exo (Figure 3C). Pyroptosis assay results show that H/R significantly increased LDH release, which was significantly suppressed by supplementation of NC-exo and mimic-exo (Figure 3D). Hoechst 33342/PI staining showed that H/R significantly increased the PI-positive cell number, which was decreased by supplementation of NC-exo and mimic-exo (Figure 3E). Supplementation of NC-exo and mimic-exo also significantly attenuated H/R-induced upregulation of NLRP3 (Figure 3F), upregulation of activated casp-1(p20) and GSDMD-N (Figure 3G), and elevation of IL-1β and IL-18 (Figure 3H). In comparison to NC-exo, mimic-exo had a more obvious effect, and inhibitor-exo showed less effect. The findings suggest that hucMSC-exo protects against H/R-caused pyroptosis in AC16 cells via miR-100-5p.

### miR-100-5p Negatively Regulates FOXO3/NLRP3

**FIGURE 4:**
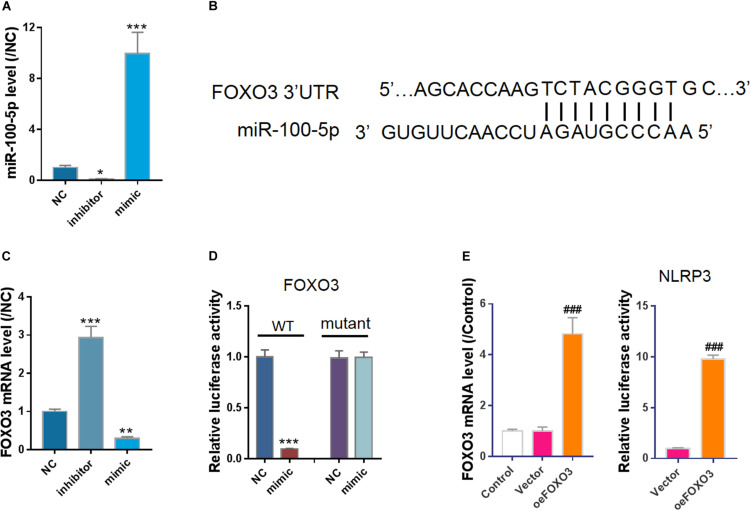
FOXO3, a transcription factor of NLRP3, was a potential target of miR-100-5p. **(A)** Control miRNA (NC), inhibitor (inhibitor), or mimic (mimic) was transfected into cells. qPCR results showed miR-100-5p was successfully silenced or overexpressed. **(B)** miR-100-5p potential binding site in FOXO3 3’-UTR predicted by miRWalk. **(C)** Overexpression of miR-100-5p suppressed FOXO3 expression. **(D)** Bioluminescence assay indicated miR-100-5p bound to FOXO3 3’-UTR to suppress its transcription. **(E)** Overexpression of FOXO3 enhanced NLRP3 promoter activity. *P < 0.05, **P < 0.01, ***P < 0.001 vs. NC; ^###^*P* < 0.001 vs. vector.

To investigate how miR-100-5p is involved in the protective effect of hucMSC-exo, negative control miRNA (NC), miR-100-5p-inhibitor, or miR-100-5p-mimic was transfected into HEK293 cells (Figure 4A). Using bioinformatics analysis, we found a potential binding site of miR-100-5p on FOXO3 3’-UTR (Figure 4B). *In vitro* cell study shows that FOXO3 mRNA expression was suppressed by miR-100-5p-mimic but enhanced by miR-100-5p-inhibitor (Figure 4C). A bioluminescence assay further confirmed that miR-100-5p bound to FOXO3 3’-UTR to suppress its transcription (Figure 4D). Furthermore, as FOXO3 is a well-known transcription factor, a luciferase assay was performed, and the results showed that overexpression of FOXO3 enhanced the promoter activity of NLRP3 (Figure 4E). Collectively, we show that miR-100-5p binds to 3’-UTR of FOXO3 to suppress its transcription, leading to the downregulation of NLPR3.

### FOXO3 Overexpression Reverses the Protective Effects of hucMSC-exo in H/R Injury

**FIGURE 5:**
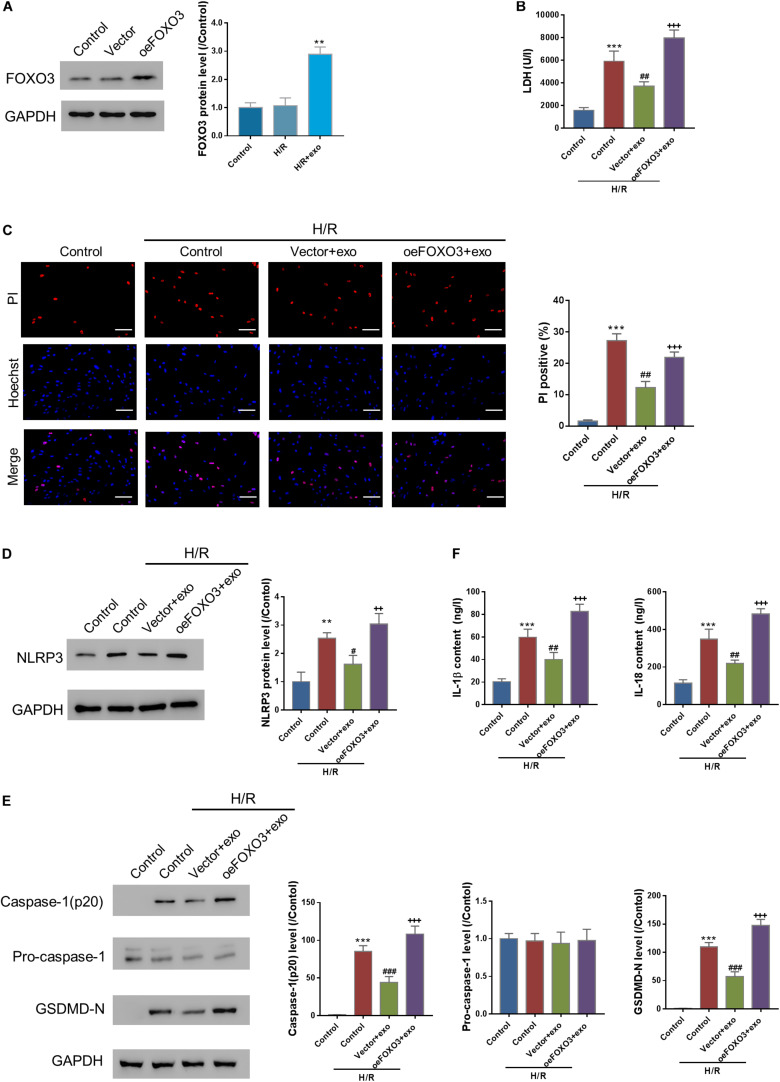
FOXO3 overexpression reversed the protective effects of hucMSC-exo in H/R injury. **(A)** FOXO3 was successfully overexpressed in AC16 cells. **(B–F)** AC16 cells overexpressing FOXO3 or vector were incubated with hucMSC-exo for 24 h, followed by H/R. LDH release **(B)**, pyroptosis rate (**C**, Hoechst 33342/PI staining, Scale bar: 100 μm), protein expression of NLPR3, activated caspase-1(p20), pro-Caspase-1, and GSDMD-N (**D,E**, Western blot analysis), and the release of IL-1β and IL-18 (**F**, ELISA analysis) were determined. ^∗∗^*P* < 0.01, ^∗∗∗^*P* < 0.001 vs. control; ^#^*P* < 0.05, ^##^*P* < 0.01, ^###^*P* < 0.001 vs. control + H/R; ^++^*P* < 0.01, ^+++^*P* < 0.001 vs. vector + exo.

Next, the effects of FOXO3 overexpression on H/R injury were investigated. FOXO3 was first overexpressed in AC16 cells (Figure 5A). Then, AC16-oeFOXO3 or AC16-vector cells were incubated with hucMSC-exo for 24 h, followed by H/R challenge. As expected, the protective effects of hucMSC-exo were all blocked by FOXO3 overexpression as indicated by the analysis, LDH release and PI positive cells, the expression of NLPR3, activated casp-1, and GSDMD-N as well as the release of IL-1β/IL-18 (Figures 5B–F). These findings suggest that hucMSC-exo protects AC6 cells against H/R-induced injury in a FOXO3-dependent manner.

## Discussion

Pyroptosis, or caspase 1–dependent cell death, is a form of inflammatory programmed cell death pathway activated by inflammatory cytokines, including caspase-1, caspase-5, or caspase-11 (Man et al., 2017). Increasing evidence suggests that pyroptosis is involved in numerous diseases, including cardiovascular diseases (Walle and Lamkanfi, 2016). H/R injury is a widely accepted *in vitro* model of I/R (Li and Jackson, 2002). In the current study, we observed that H/R caused significant injury to AC16 cardiomyocytes manifested by decreased cell viability and enhanced pyroptosis. We also find that H/R caused significant elevation of activated caspase-1, GSDMD-N, and IL-1β/IL-18, which was triggered by NLRP3 inflammasome activation (Walle and Lamkanfi, 2016).

MSC-exo are shown to be effective in treating cell death in myocardial infarction (Zhu et al., 2018). We find in this study that hucMSC-exo treatment protects against H/R-induced pyroptosis in cardiomyocytes. Approximately 2200 miRNAs are reported to exist in the mammalian genome (Chakraborty et al., 2017, 2020a,b). Dysregulation of miRNAs has been linked to numerous diseases, including myocardial infarction (Chakraborty et al., 2020b), and miRNA-based therapeutics have shown clinically significant benefits for various diseases (Chakraborty et al., 2020b). Previous studies reveal the functions of exosomal miR-100-5p in different diseases. For example, exosomes from cisplatin-resistant lung cancer cells have low expression of miR-100-5p, which could alter cisplatin sensitivity of other lung cancer cells in a miR-100-5p-dependent manner (Qin et al., 2017). A recent study reports that infrapatellar fat pad MSCs-derived exosomes protect articular cartilage from damage via delivering exosomal miR-100-5p (Wu et al., 2019). We find in this study that exosomes from hucMSC transfected with miR-100-5p-inhibitor had little effect on H/R-induced pyroptosis, and H/R induced increase of NLPR3 expression, activated caspase-1, GSDMD-N, IL-1β, and IL-18. These data indicate that miR-100-5p is crucial in the protective role of hucMSC-exo on H/R-treated AC16 cells. Our study, consistently with previous reports, suggests that the exosomal transfer of miR-100-5p is an important mechanism for the regulation of recipient cell functions. Additionally, H/R treatment suppressed the expression of miR-100-5p. It is reported that H/R could elevate intracellular reactive oxygen species (ROS) in cardiomyocytes (Park et al., 2011). Considering that expression of miRNAs could be altered by agents that induce oxidative stress (Magenta et al., 2013), we suppose that the expression of miR-100-5p is regulated by H/R-induced ROS, and further investigation is needed.

FOXO3 is a member of the FOXO subclass of transcription factors, which play an important role in a variety of biological processes, including apoptosis, proliferation, and invasiveness (Stefanetti et al., 2018). Several miRNAs are reported to regulate the expression of FOXO3 in various cell types via directly binding to 3’-UTR, such as miR-155 in liver cancer cells (Liao et al., 2018), miR-10b-3p in esophageal squamous cell carcinoma cells (Lu et al., 2018), and miR-34a in macrophages (Song et al., 2017). In this study, we prove that miR-100-5p suppresses the transcription of FOXO3 via binding to 3’-UTR, leading to subsequent NLRP3 inhibition.

FOXO3 overexpression is implicated in a variety of patho-biological processes. For example, FOXO3 overexpression is shown to counteract the miR-223 inhibitory effect on apoptosis in active tuberculosis patients (Xi et al., 2015). Abid et al. (2005) show that FOXO3 overexpression inhibited vascular smooth muscle cell proliferation and neointimal hyperplasia. Another study indicates that overexpression of FOXO3 results in suppression of growth and survival of breast cancer cells (Zou et al., 2008). Our current study shows that overexpression of FOXO3 ameliorates the protection effects of hucMSC-derived exosomes, suggesting that FOXO3 is of great importance in H/R-caused inflammasome activation, cytokine release, and pyroptosis.

## Conclusion

**FIGURE 6:**
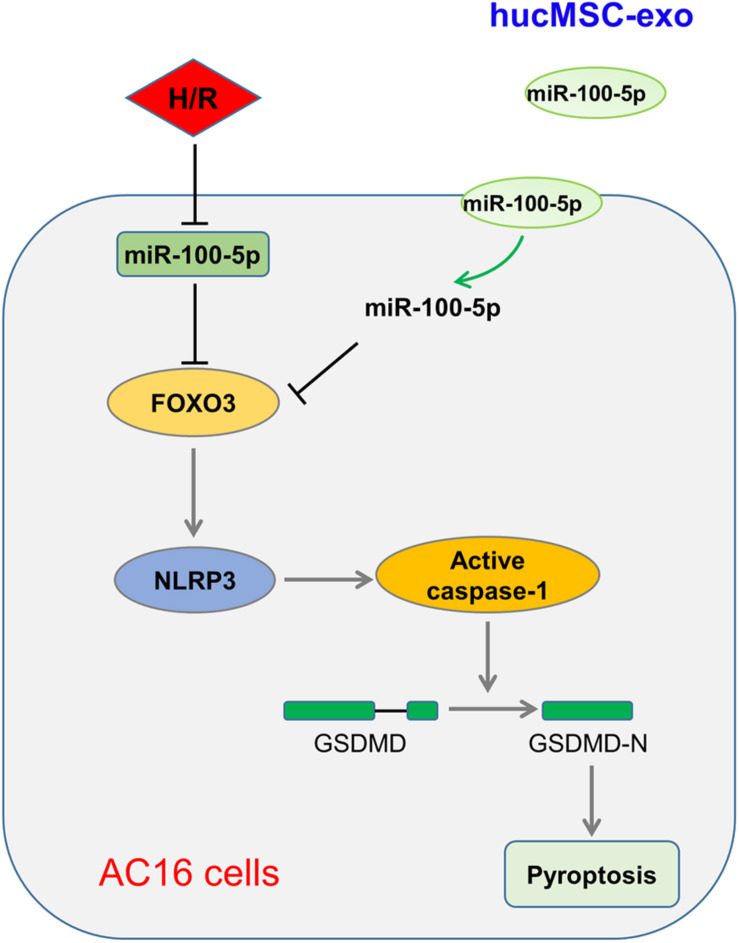
Schematic illustration of hucMSC-exo protected H/R-challenged AC6 cells via the miR-100-5p/FOXO3/NLRP3 pathway.

Taken together, our study shows that enriched miR-100-5p in hucMSC-exo suppresses FOXO3 expression to inhibit NLRP3 inflammasome activation, suppresses cytokine release, and represses pyroptosis, therefore protecting cardiomyocytes from H/R-induced injury (Figure 6). In addition, some limitations are presented in this study, such as lack of detailed mechanisms and *in vivo* animal experiments, which will be investigated in further research. Nevertheless, this study suggests the potential clinical application of hucMSC-exo for I/R injury.

## Data Availability Statement

The raw data supporting the conclusions of this article will be made available by the authors, without undue reservation, to any qualified researcher.

## Ethics Statement

The studies involving human participants were reviewed and approved by the ethical review board of Tongji Hospital of Tongji University. The patients/participants provided their written informed consent to participate in this study.

## Author Contributions

CL and ML: conception and design. YS: provision of study materials or patients. CL, YL, and HX: collection and assembly of data. CL, JH, and FC: data analysis and interpretation. CL, YL, and ML: manuscript writing. All authors final approval of manuscript.

## Conflict of Interest

The authors declare that the research was conducted in the absence of any commercial or financial relationships that could be construed as a potential conflict of interest.
