# Supplementary material for: Exosomes of Human Umbilical Cord MSCs Protect Against Hypoxia/Reoxygenation-Induced Pyroptosis of Cardiomyocytes via the miRNA-100-5p/FOXO3/NLRP3 Pathway
Source: Front Bioeng Biotechnol. 2021 Jan 15;8:615850. doi: 10.3389/fbioe.2020.615850 (PMC7844314; doi:10.3389/fbioe.2020.615850)
Supplement: Supplementary file 1 [file Data_Sheet_1.docx]

**Table S1.** Antibodies for flow cytometry analysis.

| Antibody name | Company | Catalog # |
| --- | --- | --- |
| CD90 Monoclonal Antibody , FITC | eBioscience™ | 11-0909-42 |
| CD44 Monoclonal Antibody , FITC | eBioscience™ | 11-0441-82 |
| CD105Monoclonal Antibody , PE | eBioscience™ | 12-1057-42 |
| CD11b Monoclonal Antibody , PE | eBioscience™ | 12-0118-42 |
| CD34 Monoclonal Antibody , PE | eBioscience™ | 12-0349-42 |
| CD45 Monoclonal Antibody , FITC | eBioscience™ | 11-0459-42 |

**Table S2.** Primer sequence.

| Name | Primer sequences |
| --- | --- |
| miR-100-5p | RT primer:  5’-GTCGTATCCAGTGCAGGGTCCGAGGTATTCGCACTGGATACGACCACAAG- 3’ |
|  | Forward: 5’-GCGAACCCGTAGATCCGAA-3’ |
|  | Reverse: 5’-AGTGCAGGGTCCGAGGTATT-3’ |
| U6 small nuclear 1 | Forward: 5’- CTCGCTTCGGCAGCACA -3’ |
|  | Reverse: 5’- AACGCTTCACGAATTTGCGT -3’ |
| NLRP3 | Forward: 5’- CCTGGAGGATGTGGACTTG -3’ |
|  | Reverse: 5’- GGTCTGCCTTCTCTGTCTG -3’ |
| FOXO3 | Forward: 5’- CCAGGGTAAAGTCAAGTG -3’ |
|  | Reverse: 5’- GCAGGGTCTCAACATAAG -3’ |
| GAPDH | Forward: 5’- AATCCCATCACCATCTTC -3’ |
|  | Reverse: 5’- AGGCTGTTGTCATACTTC -3’ |

**Table S3.** Antibodies for Western blot.

| Antibody | Company | Catalog # | Dilution |
| --- | --- | --- | --- |
| NLRP3 | Abcam | Ab214185 | 1:2000 |
| CD9 | Abcam | Ab92726 | 1:2000 |
| CD63 | Abcam | Ab134045 | 1:5000 |
| Alix | Abcam | ab88743 | 1:1000 |
| GSDMD-N | Novus | NBP2-80427 | 1:1000 |
| Caspase-1(p20) | Proteintech | 22915-1-AP | 1:1000 |
| Pro-Caspase-1 | Abcam | Ab179515 | 1:1000 |
| GAPDH | Proteintech | 60004-1-1G | 1:5000 |
